# Successful treatment of refractory IgA vasculitis with tofacitinib

**DOI:** 10.1016/j.jdcr.2022.09.030

**Published:** 2022-10-11

**Authors:** Yi-Fei Xu, Zi-Qi Li, Wan-Shan Yang, Xiao-Wei Shi, Guang-Ming Han

**Affiliations:** Department of Dermatology and Rheumatology, Dermatology Hospital of Southern Medical University, Guangzhou, China

**Keywords:** IgA vasculitis, tofacitinib

## Introduction

IgA vasculitis, formerly known as Henoch-Schönlein purpura, is a small vessel vasculitis that is characterized by non-thrombocytopenic purpura.^1^ Very little is understood about the pathogenesis of IgA vasculitis except that the IgA-immune complex is found to deposit in the skin and, hence, the change in nomenclature.^2^

In general, most cases of children with IgA vasculitis (aged <10 years), particularly those that present only skin involvement, are usually self-limiting and do not require specific treatment. If pediatric patients present with a bullous or necrotic rash or IgA vasculitis nephritis, as well as adult patients, then the treatment may be required. However, to our knowledge, there are no recommendations for the treatment of IgA vasculitis based on double blind, placebo-controlled trials. Although most patients with IgA vasculitis effectively respond to common drugs, such as corticosteroids, dapsone, or azathioprine, a minority of these patients failed to respond to them; therefore, highlighting the need for additional treatment options for these refractory cases.^3^

Tofacitinib is a Janus kinase inhibitor that has been reported to treat successfully a case of refractory vasculitis: polyarteritis nodosa.^4^ However, to our knowledge, there is no report describing its efficacy in IgA vasculitis. Herein, we describe a case of refractory IgA vasculitis successfully treated with tofacitinib.

## Case report

**Fig 1:**
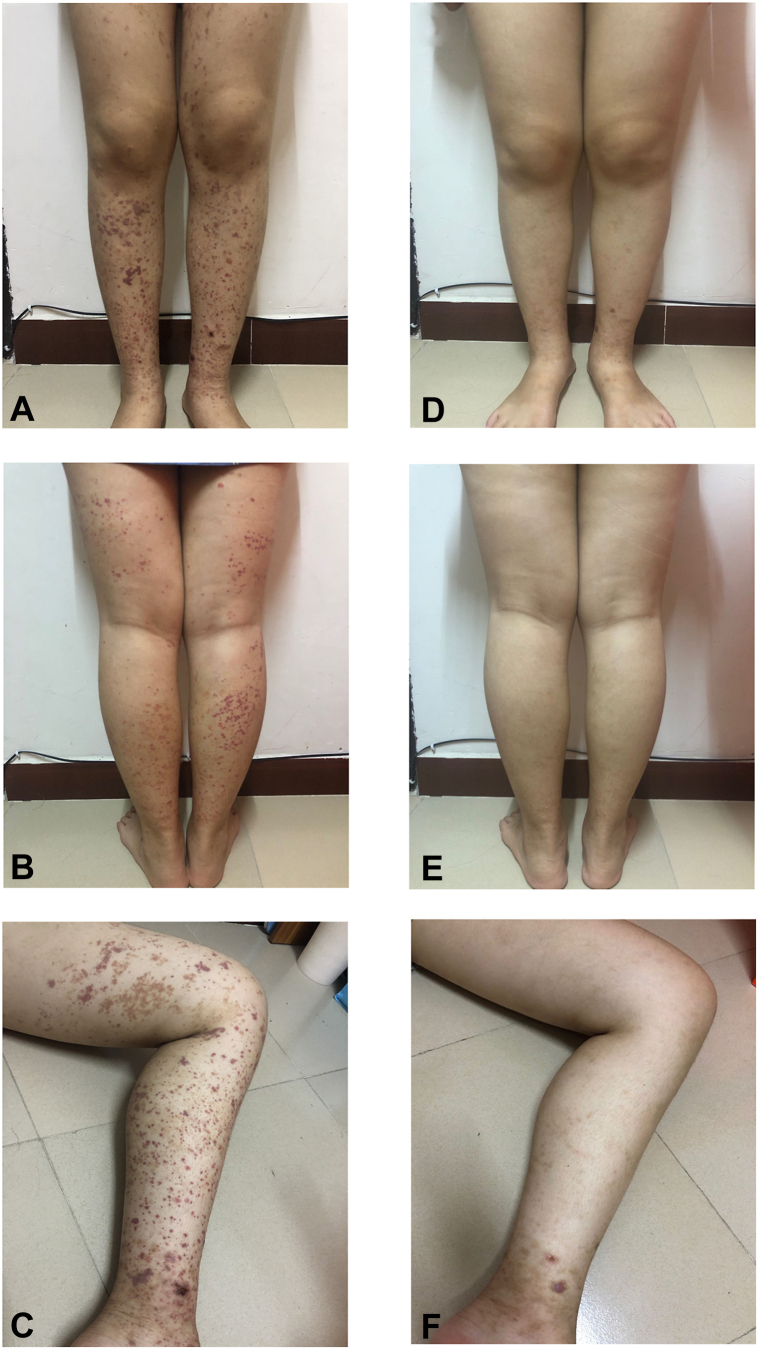
Clinical Photographs before and after treatment with tofacitinib for 2 weeks. ***A*,** purpura on the front of both the legs before treatment with tofacitinib; ***B***, purpura on the back of both the legs before treatment with tofacitinib; ***C*,** inside of the left leg before treatment with tofacitinib; ***D*,** purpura on the front of both the legs after treatment with tofacitinib for 2 weeks; ***E*,** purpura on the back of both the legs after treatment with tofacitinib for 2 weeks; and ***F*,** the inside of left leg after treatment with tofacitinib for 2 weeks (clinical photographs were provided by the patient).

A 15-year-old girl presented with palpable purpura on both lower legs, with occasional abdominal pain and arthralgia of knees, without hematuria and proteinuria (Fig 1, *A* to *C*). Over the previous 6 weeks, she was diagnosed with a progressive IgA vasculitis in the local hospital, and was initially treated with a 2-week course of oral corticosteroids (30 mg/d), and then sequentially treated with thalidomide (100 mg/d) for 2 weeks and dapsone (2 mg/kg/d) for another 2 weeks.

**Fig 2:**
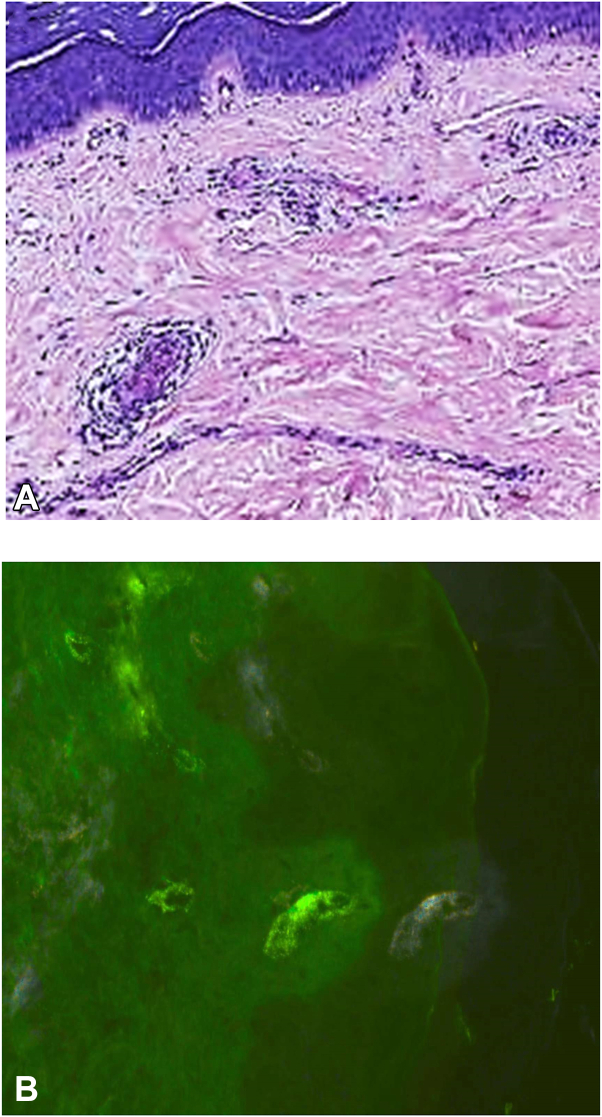
Histopathologic and direct immunofluorescence findings of the skin. **A,** Leukocytoclastic vasculitis of the small blood vessels in the dermis (Hematoxylin-eosin stain; original magnifications: ×40); and **B,** Direct immunofluorescence: granular IgA deposits seen in the walls of the small blood vessels in the dermis.

Despite therapy, her palpable purpura worsened. At this point, she was evaluated by our service. On physical examination, the patient was afebrile. Vital signs: temperature was 37.5 °C, pulse was 66 times/min, and regular, blood pressure was 100/76 mmHg. Skin examination revealed palpable purpura on both of the lower legs (Fig 1, *C*). Abdominal examination was normal. She had a full range of motion in all joints without any swelling or synovitis. Pertinent laboratory tests, such as complete blood cell count and coagulation tests, had negative results. Screening assay results for interferon-γ release for tuberculosis and hepatitis B and C were negative. A skin biopsy histopathologic examination showed leukocytoclastic vasculitis (Fig 2, *A*), and direct immunofluorescence showed granular IgA deposits in the walls of the small blood vessels in the dermis (Fig 2, *B*). Subsequently, she received a diagnosis of IgA vasculitis. Due to no effect of these common drugs, she was diagnosed with refractory IgA vasculitis.

After obtaining informed consent from the patient, we initiated tofacitinib therapy (10 mg/d). Seven days after treatment, palpable purpura was substantially improved. Two weeks after treatment, purpura disappeared (Fig 1, *D* to *F*). Then, the patient was tapped to 5-mg once daily doses of tofacitinib. At the time of writing this report, the patient was receiving tofacitinib therapy 5 mg once daily for 1 month and was in a state of remission with no adverse events.

## Discussion

In this case report, we treated a patient with refractory IgA vasculitis with tofacitinib and we found that it inhibited disease progression.

Due to no effect of common drugs, such as corticosteroids, thalidomide, and dapsone, she was treated with tofacitinib. It inhibited disease progression clinically and safely maintained remission without increasing the risks of infections.

The underlying mechanism of the action of tofacitinib on refractory IgA vasculitis remains unclear. However, compared to healthy controls, the levels of proinflammatory cytokines, such as interleukin 6 (IL-6), IL-8, and tumor necrosis factor-alpha, increase in the sera of patients with IgA vasculitis.^5^ Tofacitinib is a Janus kinase inhibitor that is approved for the treatment of inflammatory rheumatoid and bowel diseases.^6^ It inhibits the signal transducers and activators of transcription pathways that regulate signaling by cytokines, including tumor necrosis factor-alpha, IL-6, and IL-17.^7^ Although it inhibits the IL-6 pathway, tofacitinib can successfully treat a case of refractory polyarteritis nodosa.^4^ Similar to patients with polyarteritis nodosa, our patient with IgA vasculitis is currently receiving tofacitinib therapy 5 mg once daily for 1 month and is in a state of remission with no adverse events.

This case indicates that tofacitinib is a potential therapeutic drug for the treatment of refractory IgA vasculitis. However, it cannot exclude the disease resolving on its own or the addition of tofacitinib being coincidental. Therefore, further larger clinical trials are needed to confirm our findings of the safety and efficacy of tofacitinib in IgA vasculitis.

## Conflicts of interest

None disclosed.
